# Health economics perspective of fesoterodine, tolterodine or solifenacin as first-time therapy for overactive bladder syndrome in the primary care setting in Spain

**DOI:** 10.1186/1471-2490-13-51

**Published:** 2013-10-21

**Authors:** Antoni Sicras-Mainar, Javier Rejas, Ruth Navarro-Artieda, Alba Aguado-Jodar, Amador Ruiz-Torrejón, Jordi Ibáñez-Nolla, Marion Kvasz

**Affiliations:** 1 Directorate of Planning, Badalona Serveis Assistencials SA, Calle Gaietà Soler, 6-8 entlo, CP 08911 Badalona, Barcelona, Spain; 2 Health Economics and Outcomes Research, Pfizer SLU, Alcobendas, Madrid, Spain; 3 Medical Information Department, Hospital Germans Trias i Pujol, Badalona, Spain; 4 CAP Sagrada Familia, Barcelona, Spain; 5 Primary Health Care Directorate, 1ib-Salut, Mallorca, Spain; 6 Directorate of Badalona Serveis Assistencials SA, Hospital Municipal de Badalona, Badalona, Barcelona, Spain; 7 Health Economics and Outcomes Research, Pfizer PIO, Paris, France

**Keywords:** Antimuscarinics, Overactive bladder, Costs, Primary care setting, Health resources

## Abstract

**Background:**

Overactive bladder (OAB) is associated with high healthcare costs, which may be partially driven by drug treatment. There is little comparative data on antimuscarinic drugs with respect to resource use and costs. This study was conducted to address this gap and the growing need for naturalistic studies comparing health economics outcomes in adult patients with OAB syndrome initiating treatment with different antimuscarinic drugs in a primary care setting in Spain.

**Methods:**

Medical records from the databases of primary healthcare centres in three locations in Spain were assessed retrospectively. Men and women ≥18 years of age who initiated treatment with fesoterodine, tolterodine or solifenacin for OAB between 2008 and 2010 were followed for 52 weeks. Healthcare resource utilization and related costs in the Spanish National Health System were compared. Comparisons among drugs were made using multivariate general linear models adjusted for location, age, sex, time since diagnosis, Charlson comorbidity index, and medication possession ratio.

**Results:**

A total of 1,971 medical records of patients (58.3% women; mean age, 70.1 [SD:10.6] years) initiating treatment with fesoterodine (n = 302), solifenacin (n = 952) or tolterodine (n = 717) were examined. Annual mean cost per patient was €1798 (95% CI: €1745; €1848). Adjusted mean (95% bootstrap CI) healthcare costs were significantly lower in patients receiving fesoterodine (€1639 [1542; 1725]) compared with solifenacin (€1780 [€1699; €1854], *P* = 0.022) or tolterodine (€1893 [€1815; €1969], *P* = 0.001). Cost differences occurred because of significantly fewer medical visits, and less use of absorbent products and OAB-related concomitant medication in the fesoterodine group.

**Conclusions:**

Compared with solifenacin and tolterodine, fesoterodine was a cost-saving therapy for treatment of OAB in the primary care setting in Spain.

## Background

Overactive bladder (OAB) is a syndrome characterized by symptoms of urinary urgency, with or without urge incontinence, often accompanied by daytime frequency and nocturia. It is caused by an overactive detrusor muscle and may be accompanied by neurological dysfunction [1-3]. The prevalence in adults ranges between 10% and 20% and increases with age [4-6]. In Europe the prevalence in the general population >18 years of age is 11.8% and is similar for men and women [4,7,8]. In Spain the population-based EPICC study estimated the prevalence of OAB to be 21.5% in adults ≥40 years of age and 38.5% in institutionalized people ≥65 years of age [9,10]. OAB has a negative effect on quality of life in affected individuals, which results from both the characteristic symptoms of OAB syndrome as well as the coping strategies frequently adopted to reduce symptom impact [11,12]. People often take extreme measures to reduce urinary frequency and episodes of incontinence, which significantly impacts their physical health, vitality, social life, emotional state and functionality [13-15]. Many individuals do not seek professional help for OAB symptoms and only a quarter receive treatment [5,16,17].

Pharmacological treatment of OAB aims to decrease involuntary contractions of the detrusor muscle of the bladder by blocking the bladder receptors with antimuscarinic drugs [18-21]. Efficacy, safety, and tolerability [22-26] as well as improved health-related quality of life [27] have been demonstrated in clinical studies for the antimuscarinics fesoterodine, tolterodine and solifenacin. However little is known about how these findings from clinical trials extrapolate to the heterogeneous populations seen in primary practice. Several studies indicate that real-world adherence is low and is typically attributed to a perceived lack of efficacy or tolerability issues [28,29].

OAB is associated with high direct healthcare costs [30-32]. Factors contributing to the cost of OAB include drug treatment, physician visits, hospitalizations and laboratory tests. Evidence comparing antimuscarinic drugs with respect to resource use and costs is limited, and totally lacking from a Spanish National Health System (NHS) perspective. To address this gap and the growing need for naturalistic multicenter studies, this study was conducted to compare healthcare resource utilization and related costs in patients initiating treatment with fesoterodine, tolterodine, or solifenacin for the treatment of OAB syndrome in the primary care setting in a representative sample of the Spanish NHS.

## Methods

### Design and objectives

In this observational, multicenter, post-marketing, longitudinal, retrospective study, existing medical records (computerized databases with de-identified data) of outpatients and inpatients with a diagnostic code of OAB in three different locations in Spain were extracted and reviewed.

The main objectives of the study were to compare healthcare resource utilization related to the clinical management of OAB syndrome and corresponding costs in patients treated with fesoterodine, tolterodine, or solifenacin for the first time in the primary care setting in Spain’s NHS.

### Study population

The study population consisted of patients from three geographical areas of two autonomous regions (Catalonia [Badalona and Barcelona locations] and the Balearic Islands [Mallorca]). These regions were selected because of the existence of available healthcare provider databases and because one region (Catalonia) is continental and large while the other (the Balearic Islands) is insular and small, meaning that the varied geographical composition of the country is reflected in the study and thus may be considered representative of Primary Care settings in Spain. Eighteen primary health care centres (PHC), 7 in Badalona, 2 in Barcelona and 9 in Mallorca, and their corresponding referral hospitals (three) participated in the study. The population assigned to these centres is mostly urban and industrial and low- to middle-class socioeconomic status. The follow-up period was 52 weeks from the initiation date of antimuscarinic treatment (index date).

Eligible men and women were ≥18 years of age who had not received any antimuscarinic therapy for ≥1 year before the index date; who sought medical care and initiated treatment with fesoterodine, tolterodine or solifenacin during the recruitment period (1 January 2008 to 31 December 2010); who received treatment within the designated healthcare system area; who were followed up regularly as demonstrated by ≥2 computerized health records in the corresponding health area; and who participated in a prescription drug program. Patients who transferred to other primary care centres, regions, or health areas; who were treated simultaneously with ≥2 antimuscarinic drugs during the study period; or who were permanently institutionalized were excluded. Sampling of medical records was exhaustive without reposition among those meeting eligibility criteria for the study.

### Database information

Clinical data on patients treated with fesoterodine, tolterodine or solifenacin (Anatomical Therapeutic Chemical Classification System [33]) were obtained from claim databases using the same informatics system for filing and handle health data (ONMI-AP software). These databases are owned by Badalona Serveis Assistencials (BSA), a healthcare provider in Badalona (Barcelona), Sagrada Familia Primary Care Health Centre in Barcelona Spain, and the Primary Health Care Directorate of ib-Salut, the healthcare provider for Mallorca. The choice of specific medicine and dose was determined by the patients’ physicians as part of routine primary care. Data on prescribed doses of fesoterodine (4 and 8 mg), solifenacin (5 and 10 mg) and tolterodine (2 and 4 mg) and treatment time (in weeks) during follow-up were recorded in the database.

### Compliance

Compliance, as defined by the International Society for Pharmacoeconomics and Outcomes Research working group, was computed using the medication possession ratio (MPR) [34]. The MPR is measured from first to last prescription and represents the number of days of medication supplied divided by number of days the patient was followed (starting with the index date) [35]. The MPR is viewed as a proxy of compliance because compliance may be overestimated by simply summing the number of days’ supply; patients may refill medication before finishing the current fill.

### Definition of overactive bladder and co-morbidities included in the study

The diagnosis of OAB was confirmed in enrolled patients using the International Classification of Primary Care (ICPC-2), component 7; diseases and health problems [36] (U13), and hospital and emergency room discharge coding, according to the International Classification of Diseases, 9th Revision, Clinical Modification, ICD-9-CM (596.51). The main study variables were: age (continuous and ranges), sex, occupational status (active, retired), time from diagnosis until treatment initiation and personal history (ICPC-2) of hypertension (K86, K87), diabetes mellitus (T89, T90), dyslipidemia (T93), obesity (T82), smoking (P17), alcoholism (P15, P16), all types of organ failure (cardiac, hepatic and renal), cerebrovascular accident (K90, K91, K93), chronic obstructive pulmonary disease (R95, chronic airflow obstruction), bronchial asthma (R96), dementia or memory disorders (P70, P20), neurological diseases: (Parkinson’s disease [N87], epilepsy [N88], multiple sclerosis [N86] and other neurological diseases [N99]), depressive syndrome (P76) and malignant neoplasms (all types, A79, B72-75, D74-78, F75, H75, K72, L71, L97, N74-76, R84-86, T71-73 , U75-79, W72-73, X75-81, Y77-79). The Charlson comorbidity index [37] was used to summarize each patient’s comorbidity status as well as the number of chronic co-morbidities.

### Resource use and cost analysis

Healthcare costs for this study were defined as all OAB-related costs, including medical care visits (primary care, specialty care [urology and gynaecology] and emergency room), days of hospitalization, and all OAB-related diagnostic or therapeutic tests (with or without urinary incontinence) performed by health professionals. Costs were expressed as the annual mean cost per patient (unit/cost) for fesoterodine, tolterodine or solifenacin treatment. A full list of resources collected and associated unit costs are shown in Table 1. Year 2011 prices were used to compute unit costs. Use of absorbent products for urinary incontinence and concomitant medications associated with the potential clinical consequences of OAB were included in estimated costs. Concomitant drugs of interest included antidepressants, anxiolytics, hypnotics, antibiotics, and antiseptics for urinary tract and/or skin infections (systemic or topical) and laxatives used for the treatment of antimuscarinic-related constipation. Costs of acute, chronic and on-demand prescriptions were calculated using the retail price per pack at the time of prescribing. Direct non-health costs (disease-associated out-of-pocket costs paid by patients and their families) were not included in the study because these are not captured in the database, and the database does not provide direct access to patients. Overall costs by drug were assessed for a 52-week follow-up period. Baseline healthcare resource use and associated costs were also determined for 1 year prior to antimuscarinic initiation.

**Table 1:** Unit costs of resources utilized

**Health and non-health resources**	**2011 Unit costs (€)**
Medical visits	
Primary care	23.19
Emergency room	117.53
Specialist	104.41
Complementary tests*	
Laboratory tests^†^	22.30
Conventional radiology^‡^	18.50
Diagnostic/therapeutic tests*	37.12
Drug prescription^§^	Retail^‖^
Hospitalization (one day in a general ward)	320.90

Information sources: study site cost accounting and Spanish National Institute of Statistics.

*Includes ultrasound scans or other diagnostic imaging tests and electrocardiograms.

^†^Includes blood count, biochemistry, lipid panel, urinalysis, thyroid function.

^‡^ Includes X-ray measurements.

^§^ Includes the costs of antimuscarinic drugs, absorbent products and concomitant medications (antidepressants, anxiolytics, antibiotics, anti-infective drugs, laxatives and dermatologic products for skin infections).

^‖^Retail: retail price + value added tax (for year 2011).

### Data confidentiality and ethics statement

This was a retrospective observational study using existing data on patients included in a claim database. Data confidentiality was respected at all times by means of de-identification thus ensuring anonymity according to Spanish Organic Law (Law 15/1999 of 13 December on the Protection of Personal Data). Thus no data which could reveal patient identity was extracted. The database was then prepared and closed for statistical analysis, not allowing further manipulation of data. The study, its design and procedures was classified by the Spanish Medicines Agency (AEMPS) as a Post-Authorisation Study – Other Designs (EPA-OD) trial type and was subsequently approved by the Clinical Research Ethics Committee of Hospital Clinic I Provincial of Barcelona. Based on its design, it was exempt under Spanish law from requiring written informed consent (Law 29/2006 of 26 July and Royal Decree 1344/2007 of 11 October; see http://www.aemps.gob.es/investigacionClinica/medicamentos/estudiosPostautorizacion.htm).

### Statistical analysis

In this population-based study, the sample size was calculated according to an expected prevalence of OAB of 15% in the country, assuming a random error of 5% and an estimated precision of 2%. The study required medical records in a 1:2:2 proportion for fesoterodine, solifenacin and tolterodine, respectively, to match the expected distribution of use of these drugs on the Spanish market. A minimum of 300:600:600 medical records (one record per patient) were necessary to be able to detect a difference in healthcare costs of €260 (standard error, 71) between two drugs with an α level of <0.05 (with degree of freedom corrected after applying a Bonferroni adjustment for multiple comparisons) and a minimum power of 80%.

Computerized medical records were assessed for frequency distributions as well as possible errors in registration or coding. Descriptive univariate statistical analyses were performed, using the mean, median, standard deviation and 95% confidence intervals (CI) for parametric variables and the median and interquartile ranges (25 and 75 percentiles of the distribution) for non-parametric variables, after verifying the normality of the distribution with the Kolmogorov-Smirnov test. Two-sample comparisons were performed for all variables with location as a grouping variable to confirm homogeneity of data across study locations (Badalona, Barcelona and Mallorca). Although no clinically relevant differences were noted, location was included as a covariate in all subsequent analyses. Persistence with antimuscarinic treatment was initially analyzed descriptively by calculating the proportion of patients who remained on treatment during the 52-week period following the index date.

Resource utilization and costs were compared using robust analysis of variance (ANOVA) tests (Welch test and Brown-Forsythe test with a subsequent pairing of contrasts using the Games-Howell test) and the chi-square test, and depending on the distribution of the data, a bivariate analysis, without controlling for possible confounding variables. Resource utilization and the associated costs were compared according to the recommendations of Thompson and Barber [38], using a multivariate general linear model controlling for the following covariates: location, age, sex, MPR, time since diagnosis and disease burden. Disease burden included the number of comorbid diagnoses and the Charlson comorbidity index. Pairwise comparisons were adjusted using the procedure for estimating marginal means, applying the Bonferroni correction to estimate the *P* value of statistical significance. The data are presented as adjusted mean differences between treatments with corresponding 95% CIs calculated using re-sampling techniques (bootstrapping) corrected for bias, given the non-normal distributions of the variables with respect to resource utilization and costs. The analysis was performed using the statistical package SPSS for Windows version 19. Statistical significance was established as *P* < 0.05, all tests were 2-sided.

Unit drug costs were obtained from prices listed at the time of prescription, which is an approximation because drug prices may have varied during follow-up. We performed a secondary analysis by applying a 40% reduction in the cost of tolterodine and 7.5% reduction in the costs of solifenacin and fesoterodine. The 40% reduction in the price of tolterodine is justified by the recent (3^rd^ quarter 2012) loss of exclusivity of this drug; the reduction was applied to the price at the time described above. The 7.5% reduction in the price of fesoterodine and solifenacin is justified by the discount applicable to these active principles set in Royal Decree RD 8/2010 of 20 May.

## Results

**Figure 1:**
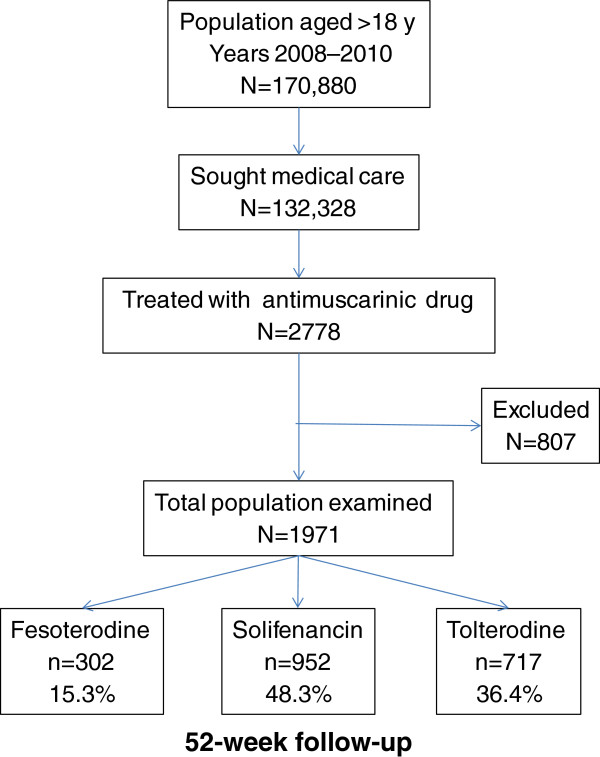
Patient record flow chart.

Of the 170,880 patients regularly seen at the study sites, 2778 patients initiated OAB treatment during the study period. Of these, 1971 met the study criteria and were enrolled (Figure 1). Of these, 952 (48.3%) were treated with solifenacin, 717 (36.4%) with tolterodine and 302 (15.3%) with fesoterodine. Patients treated with tolterodine were significantly older and included significantly more females compared with solifenacin and fesoterodine (Table 2). However disease burden, assessed by the number of comorbid diagnoses and the Charlson comorbidity index, was similar in the 3 groups. The mean time from diagnosis to initiation of antimuscarinic treatment and the MPR were significantly higher with solifenacin compared with tolterodine and fesoterodine. The mean duration of antimuscarinic treatment was numerically higher for fesoterodine, but no statistical differences were found among drugs. The most commonly prescribed doses were fesoterodine 8 mg, solifenacin 5 mg and tolterodine 4 mg. A higher percentage of patients treated with tolterodine, compared with fesoterodine or solifenacin, were prescribed a second antimuscarinic treatment during the study period (7.9% vs 4.3% and 3.6%, respectively; *P* = 0.001).

**Table 2:** Demographic and clinical characteristics by type of antimuscarinic drug

**Characteristics**	**Antimuscarinic**			
	**Fesoterodine**	**Solifenacin**	**Tolterodine**	**Overall**
	**(n = 302)**	**(n = 952)**	**(n = 717)**	***P *** **Value**
Demographics				
Mean age, years (SD)	67.5 (11.0)	69.7 (10.8)*	71.7 (9.8)*^†^	<0.001
Female, n (%)	156 (51.7)	562 (59.0)	432 (60.3)^‡^	0.036
Retired, n (%)	254 (84.1)	836 (87.8)	673 (93.8)*^†^	<0.001
Burden of comorbidity				
Associated diagnoses, n (%)	2.6 (1.7)	2.8 (1.7)	2.8 (1.5)	0.135
Charlson comorbidity index, mean (SD)	1.2 (1.3)	1.2 (1.3)	1.3 (1.4)	0.230
Score, n (%)				0.326
0–1	228 (75.5)	703 (73.8)	516 (72.0)	
2–3	50 (16.6)	166 (17.4)	120 (16.7)	
≥4	24 (7.9)	83 (8.7)	81 (11.3)	
Associated co-morbidities, n (%)				
Hypertension	142 (47.0)	494 (51.9)	413 (57.6)^‡^	0.004
Diabetes	62 (20.5)	233 (24.5)	178 (24.5)	0.304
Dyslipidemia	148 (49.0)	454 (47.7)	385 (53.7)^‡^	0.048
Obesity	66 (21.9)	204 (21.4)	147 (20.5)	0.854
Active smoker	44 (14.6)	140 (14.7)	69 (9.6)^†‡^	0.006
Alcoholism	13 (4.3)	22 (2.3)	16 (2.2)	0.124
Ischemic heart disease	34 (11.3)	114 (12.0)	77 (10.7)	0.731
Cerebrovascular accident	44 (14.6)	135 (14.2)	119 (16.6)	0.378
Organ failure	34 (11.3)	132 (13.9)	87 (12.1)	0.389
Bronchial asthma	32 (10.6)	104 (10.9)	64 (8.9)	0.393
COPD	34 (11.3)	125 (13.1)	93 (13.0)	0.685
Dementia (all types)	25 (8.3)	88 (9.2)	78 (10.9)	0.357
Neuropathies	33 (10.9)	100 (10.5)	82 (11.4)	0.833
Depressive syndrome	20 (6.6)	86 (9.0)	69 (9.6)	0.298
Malignant neoplasms	64 (21.2)	216 (22.7)	159 (22.3)	0.862
Treatment duration and dose, mean (SD)				
Time since OAB diagnosis, years	4.4 (1.8)	4.8 (1.9)^‡^	4.5 (1.8)^§^	0.023
Observed treatment duration, weeks	35.5 (17.6)	33.9 (21.4)	33.0 (21.6)	0.160
Medication possession ratio, %	94.5 (8.4)	95.4 (8.3)	94.6 (8.7)	0.053
Number of antimuscarinics^‖^	1.0 (0.2)	1.0 (0.2)	1.1 (0.3)^§^	<0.001
Antimuscarinic dose, n (%)				
2 mg			86 (12.0)	
4 mg	131 (43.4)	---	631 (88.0)	
8 mg	171 (56.6)	---	---	
5 mg	---	828 (87.0)	---	
10 mg	---	74 (13.0)	---	

COPD = chronic obstructive pulmonary disease; OAB = overactive bladder.

*P < 0.001 vs fesoterodine.

^†^*P* < 0.01 vs solifenacin.

^‡^*P* < 0.01 vs fesoterodine.

^§^*P* < 0.001 vs solifenacin.

^‖^During 52 weeks of follow-up.

No statistically significant differences were found in pre-treatment costs among treatment groups. Costs for outpatient visits, analytical tests, radiology, laboratory tests, hospitalization days and absorbent products used were not significantly different by antimuscarinic group prior to the study.

Compared with patients treated with solifenacin and tolterodine, patients treated with fesoterodine used significantly fewer antidepressants (Table 3), including selective serotonin reuptake inhibitors (SSRIs) and serotonin-norepinephrine reuptake inhibitors (SNRIs). Use of antibiotics/antiseptics was significantly lower in patients receiving fesoterodine or solifenacin compared with tolterodine (*P* < 0.001 for both). Fesoterodine was associated with decreased use of benzodiazepines/hypnotics and laxatives, although differences were not significant.

**Table 3:** Percentage of patients using OAB-related concomitant medications during follow-up by antimuscarinic drug

**Concomitant medication**	**Antimuscarinic**				
	**Fesoterodine**	**Solifenacin**	**Tolterodine**	**Overall**	**Overall**
	**(n = 302)**	**(n = 952)**	**(n = 717)**	**(N = 1971)**	***P *** Value*
Antidepressants	31.8	42.6^†^	57.9^†‡^	45.6	<0.001
Tricyclics	9.2	9.1	7.4	8.6	0.610
SSRIs	16.9	23.3^§^	35.5^†‡^	26.1	<0.001
SNRIs	18.5	26.3^‖^	37.4^†‡^	28.5	<0.001
Benzodiazepines/hypnotics	49.2	53.5	58.1^§^	54.2	0.012
Antibiotics/antiseptics	20.5	20.4	35.5^†‡^	25.2	<0.001
Laxatives	19.5	21.9	23.0	21.8	0.638
Dermatological products	21.5	21.8	20.2	21.2	0.847

SSRIs = selective serotonin reuptake inhibitors; SNRIs = serotonin-norepinephrine reuptake inhibitors.

*Controlling for location, age, sex, medication possession ratio, time since diagnosis and Charlson co-morbidity index.

^†^*P* < 0.001 vs fesoterodine.

^‡^*P* < 0.001 vs solifenacin.

^§^*P* < 0.05 vs fesoterodine.

^‖^*P* < 0.01 vs fesoterodine.

After adjusting for covariates, patients treated with fesoterodine had fewer outpatient medical visits of any type compared with solifenacin and tolterodine (13.2 vs. 15.1 and 16.4, respectively; *P* < 0.001), including primary care (11.6 vs. 12.9 and 14.2, *P* < 0.001), specialist (1.5 vs. 2.0 and 2.0, *P* < 0.001), and emergency room (0.1 vs. 0.2 and 0.3, *P* < 0.001) visits (Table 4). Differences in use of analytical and complementary tests, x-rays, and days of hospitalization were not significant. Patients treated with fesoterodine had significantly lower healthcare costs versus those receiving solifenacin and tolterodine: €1639 vs €1780 and €1893, respectively (*P* = 0.003; Table 5), despite significantly higher drug costs for fesoterodine (€740 vs €626 and €624; *P* < 0.001). Fesoterodine-treated patients incurred significantly lower costs for outpatient visits compared with solifenacin-treated and tolterodine-treated patients (€433 vs €533 and €563, respectively, *P* < 0.001), especially in primary care (€268 vs €300 and €329, *P* < 0.001); concomitant medication expenses were significantly lower with fesoterodine (€216 vs €305 and €335, p <0.001). Treatment differences in adjusted costs of radiology, analytic, and complementary tests and days of hospitalization were not significant. Adjusted total healthcare costs were significantly higher in patients receiving tolterodine compared with those treated with fesoterodine (*P* < 0.05; Table 5).

**Table 4:** Use of healthcare resources per patient per year according to antimuscarinic treatment in adjusted analysis*

**Resource, mean (95% CI**^**†**^**)**	**Antimuscarinic**			***P *** **Value**
	**Fesoterodine**	**Solifenacin**	**Tolterodine**	
	**(n = 302)**	**(n = 952)**	**(n = 717)**	
Medical visits, n	13.2 (12.3; 14.1)	15.1 (14.5; 15.9)^‡^	16.4 (15.5; 17.5)^‡§^	<0.001
Primary care	11.6 (10.8; 12.5)	12.9 (12.2; 13.7)^‖^	14.2 (13.2; 15.3)^‡^	0.006
Specialist	1.5 (1.4; 1.6)	2.0 (2.0; 2.1)^‡^	2.0 (1.9; 2.0)^‡^	<0.001
Emergency room	0.1 (0.1; 0.1)	0.2 (0.2; 0.2)^‡^	0.3 (0.3; 0.3)^‡¶^	<0.001
Analytical tests, n	0.8 (0.7; 0.9)	0.7 (0.7; 0.8)	0.7 (0.6; 0.8)	0.686
X-ray, n	0.3 (0.3; 0.4)	0.4 (0.3; 0.4)	0.4 (0.4; 0.5)	0.157
Complementary tests, n	0.2 (0.1; 0.3)	0.2 (0.1; 0.2)	0.2 (0.1; 0.3)	0.849
Hospital stays, days	0.1 (0.1; 0.2)	0.2 (0.1; 0.2)	0.2 (0.1; 0.2)	0.221

*Adjusting for geographic area, age, sex, medication possession ratio, time since diagnosis and Charlson co-morbidity index.

^†^95% bootstrap confidence interval bias corrected.

^‡^*P* < 0.01 vs fesoterodine.

^§^*P* < 0.05 vs solifenacin.

^‖^*P* < 0.05 vs fesoterodine.

^¶^*P* < 0.001 vs solifenacin.

**Table 5:** Adjusted* healthcare cost (€) per patient per year by antimuscarinic drug

**Resource, mean (95% CI**^**†**^**)**	**Antimuscarinic**			
	**Fesoterodine**	**Solifenacin**	**Tolterodine**	***P *** Value*
	**(n = 302)**	**(n = 952)**	**(n = 717)**	
Medical visits	433 (411; 457)	533 (515; 552)^‡^	563 (539; 585)^‡§^	<0.001
Primary care	268 (249; 290)	300 (284; 319)^‖^	329 (305; 353)^‡§^	0.005
Specialist	152 (144; 162)	212 (208; 216)^‡^	204 (200; 207)^§¶^	<0.001
Emergency room	12 (10; 16)	21 (19; 24)^‡^	30 (27; 33)^§¶^	<0.001
Analytical tests	18 (15; 20)	17 (15; 18)	16 (14; 18)	0.686
X-ray	6 (5; 8)	7 (6; 7)	8 (7; 9)	0.157
Complementary tests	7 (5; 10)	7 (5; 8)	8 (5; 10)	0.849
Hospital stays	35 (19; 59)	46 (33; 63)	53 (41; 70)	0.509
Antimuscarinic drugs	740 (690; 796)	626 (602; 652)^‡^	624 (597; 650)^‡^	<0.001
Concomitant medication^#^	216 (180; 258)	305 (274; 338)^‡^	335 (306; 366)^‡^	0.001
Absorbents	187 (135; 244)	243 (201; 290)	289 (237; 344)^‖^	0.094
Total healthcare costs	1639 (1542;1725)	1780 (1699; 1854)^‖^	1893 (1815; 1969)^‡§^	0.003

*Controlling for location, age, sex, medication possession ratio, time since diagnosis and Charlson co-morbidity index.

^†^95% bootstrap confidence interval bias corrected.

^‡^*P* < 0.01 vs fesoterodine.

^§^*P* < 0.05 vs solifenacin.

^‖^*P* < 0.05 vs fesoterodine.

^¶^*P* < 0.01 vs solifenacin.

^#^Includes OAB-related medication use (anti-depressants, benzodiazepines, hypnotics, laxatives, anti-infective drugs).

**Figure 2:**
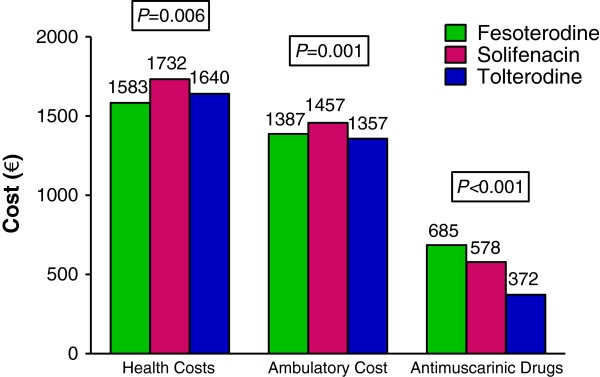
**Secondary analysis performed using actual prices for antimuscarinic drugs.** Reductions in the costs of antimuscarinic drugs were assumed for this analysis; other components of healthcare costs were kept constant. Health costs included primary (outpatient) and specialized care. Data were adjusted by location, age, sex, medication possession ratio, time since diagnosis and Charlson comorbidity index.

In a secondary analysis in which drug prices were assumed to be those currently set by the NHS (Figure 2), the trends observed in healthcare costs were similar to those described in the primary analysis. Total healthcare costs were similar for patients treated with fesoterodine and tolterodine, despite the 40% reduction in the price of tolterodine. Both fesoterodine and tolterodine were associated with significantly lower total healthcare costs than solifenacin.

## Discussion and conclusion

There are few reports detailing healthcare resource utilization and corresponding costs in routine clinical practice with antimuscarinics. This study indicates that fesoterodine treatment for OAB was associated with lower healthcare resource use than solifenacin or tolterodine treatment, and therefore lower average costs per patient, in routine clinical practice in the Spanish NHS. The treatment groups were comparable at baseline, as indicated by burden of comorbidity and duration of antimuscarinic treatment. There were no significant differences in resource use and costs incurred during the year preceding the index date; baseline costs per patient were lower because no antimuscarinics were prescribed and patients were not actively followed for OAB. This study included a large sample of medical records from three different geographic regions of Spain. Our findings did not vary by location and thus are likely generalizable throughout Spain.

Antimuscarinic cost was higher in patients treated with fesoterodine because a greater proportion of patients received the higher dose and had a longer duration of use compared with patients treated with solifenacin and tolterodine. The additional pharmacy costs found in fesoterodine-treated patients were offset by overall lower healthcare use, mostly driven by reductions in medical visits (of all types) and OAB-related concomitant medications compared with solifenacin and tolterodine. After controlling for confounding variables, fesoterodine-treated patients had a significantly lower total annual healthcare cost per patient. This represents an annual per-patient savings of approximately €141 to the Spanish NHS when compared with solifenacin and approximately €254 versus tolterodine. From a healthcare decision-making perspective, these savings are substantial in view of the number of patients with OAB in the NHS. In a secondary analysis, the total annual per-patient costs for fesoterodine were similar to those of tolterodine when the price of tolterodine was reduced by 40% to reflect current prices subsequent to the loss of patent protection in Spain. Both fesoterodine and tolterodine showed significantly lower total healthcare costs than solifenacin.

The cost advantage of fesoterodine over tolterodine may be explained by its greater efficacy, which may result from pharmacokinetic differences. Fesoterodine has greater bioavailability and therefore a greater possibility of exerting therapeutic effects. The improved efficacy of the higher fesoterodine dose (8 mg) has been established in clinical trials [22,25,39] and primary medical practice [40]; this dose was used by 57% of patients taking fesoterodine in our study.

In contrast, titration of solifenacin to the higher dose (10 mg) has not been shown to be associated with increased efficacy [41,42]; in our study, 13% of patients taking solifenacin used the 10-mg dose. A 4-mg dose of tolterodine ER is the only commercially available formulation; 12% of patients taking tolterodine received a 2-mg twice daily standard (i.e. not extended release) dose. Therefore it is possible that patients taking solifenacin and tolterodine may have received an ineffective dosage in our study. However our results under real-world conditions appear to be consistent with those observed in head-to-head comparative clinical trials. The Fesoterodine Assessment and Comparison Versus Tolterodine (FACT) trials showed that fesoterodine 8 mg is superior in efficacy to tolterodine ER 4 mg, with significantly greater reductions in episodes of urgency and urgency with incontinence and a significantly higher proportion of “dry” patients [24,25,39]. Likewise, comparative clinical trials of solifenacin versus tolterodine showed these two drugs to be similar in efficacy for the treatment of symptomatic OAB [43].

There are several limitations to this study. Some patients with OAB may have been excluded because of disease miscoding/misclassification, and some patients diagnosed as having OAB may not have the disease. The risk of disease misclassification was minimized in this study because physicians in participating locations were well trained in medical data recording and the computerized system includes regular quality control testing. Under-recording of healthcare resource use, whether an oversight by healthcare professionals or due to resource use outside of the geographical areas of interest, may have led to an underestimation of total costs. In all cases these potential biases are likely to have affected the three study drugs equally. Direct non-health costs (i.e. out-of-pocket costs paid by the patient that are not funded by the NHS) could not be assessed, because none of the three databases analyzed accounts for these costs. Out-of-pocket costs of OAB can be substantial, particularly for non-funded absorbent products. Because only 10% of the records analyzed were on occupationally active patients, an economic analysis that considers effects on productivity could not be performed. Furthermore it was not possible to assess OAB severity among patients for any of the antimuscarinic drugs prescribed; significant differences among groups could have influenced outcomes. However the fact that no significant differences in resource use and/or healthcare costs were found in the year prior to the index date suggests that symptom severity was similar among treatment groups.

This study demonstrated that in primary clinical practice in Spain, treatment of OAB with fesoterodine was a cost-saving therapy compared with solifenacin or tolterodine in the NHS system, and that the higher cost of fesoterodine therapy could be offset by lower overall healthcare resource use. Fesoterodine was associated with decreased healthcare resource utilization, including medical visits of all types as well as concomitant medications; this reduced utilization translated into significantly lower total annual healthcare costs per patient. Future studies in other healthcare settings are needed to confirm these findings.

## Competing interest

This study was sponsored by Pfizer Inc. Javier Rejas and Marion Kvasz are employees of Pfizer, S.L.U. and Pfizer PIO, respectively. Antoni Sicras was a paid consultant to Pfizer in connection with the development of this manuscript. Statistical analysis was performed by DataClinics and was funded by Pfizer Inc. All other authors declare that they have no competing interests. Editorial support was provided by Colin Mitchell, PhD, of Complete Healthcare Communications, Inc., and was funded by Pfizer Inc.

## Authors’ contributions

All authors had complete access to the data, participated in the analysis and/or interpretation of results, drafted and approved the content of the manuscript. ASM, JR and MK participated in the design and idea of the original study and in the interpretation of data and drafting the manuscript. RNA, AAJ, ART and JIN participated in collection of data and interpretation of statistical analysis results, review of manuscript and important contribution to several parts of the manuscript. All authors were responsible for literature review and extraction of references.

## Pre-publication history

The pre-publication history for this paper can be accessed here:

http://www.biomedcentral.com/1471-2490/13/51/prepub
